# Dyke‐Davidoff‐Masson Syndrome in a Female Adult: A Rare Case of Progressive Hemiparesis, Epilepsy, and Cerebral Hemiatrophy

**DOI:** 10.1002/ccr3.70732

**Published:** 2025-08-01

**Authors:** Quang Dai La, Aiman Baloch, Muhammad Ayub, Sobia Ahmed, Enoch Lue

**Affiliations:** ^1^ Futures Forward Research Institute Toms River New Jersey USA; ^2^ The Innovative STEMagazine 501(c)3 College Station Texas USA; ^3^ Department of Biology Texas A&M University College Station Texas USA; ^4^ Mekran Medical College Turbat Balochistan Pakistan; ^5^ Department of Radiology Bolan Medical Complex Hospital Quetta Pakistan; ^6^ Department of Biology Rutgers University New Brunswick New Jersey USA

**Keywords:** calvarial thickening, cerebral hemiatrophy, Dyke‐Davidoff‐Masson syndrome, hemiparesis, hyperpneumatization, seizures

## Abstract

Consideration of Dyke‐Davidoff‐Masson syndrome (DDMS) in patients with epilepsy, hemiparesis, and cognitive impairment should be taken into account. MRI plays a key role in diagnosing this rare disorder by recognizing cerebral hemiatrophy with compensatory skull and sinus hypertrophy. Early recognition can assist with symptom management and improve patient care, the prognosis.

## 1 Introduction

Dyke‐Davidoff‐Masson syndrome (DDMS) is an uncommon neurological disorder characterized by cerebral hemiatrophy with compensatory hypertrophy of the skull and sinuses [1]. The syndrome can be congenital or acquired and was first described in 1933 [1]. Congenital DDMS is thought to be caused by prenatal vascular insults such as intrauterine infections, ischemic insults, or congenital malformations [2]. Acquired DDMS, on the other hand, occurs after birth and is produced by ischemic strokes, infection, trauma, or any other underlying cause that produces chronic brain damage [3].

Neurological deficits include seizures, hemiparesis, and cognitive deficits that are often seen with DDMS [3]. Seizures are typically the most common symptom and are often refractory to treatment [4]. Hemiparesis may be progressive and is usually contralateral to the affected hemisphere [5]. The level of intellectual handicap is determined by the extent of the brain affected [6]. Neuroimaging studies are vital to distinguishing DDMS from other causes of cerebral hemiatrophy such as Rasmussen's encephalitis, Sturge–Weber syndrome, or congenital ischemic insults [3].

A 25‐year‐old woman with epilepsy, developing right‐sided hemiparesis, and intellectual impairment is the subject of this case report of DDMS. We review the radiologic and clinical characteristics, emphasizing the importance of MRI in diagnostic evaluation.

## 2 Case Presentation

A 25‐year‐old woman presented to our clinic with a history of chronic seizures only partially managed by antiepileptic drugs. Over the past few years, the patient has experienced progressive cognitive decline and the insidious onset of right‐sided weakness. No previous neuroimaging history was present.

Upon examination, the patient had hyperreflexia, elevated muscular tone, and right‐sided hemiparesis. Gait analysis showed a spastic pattern, and the right hand and foot had minor contractures. The patient had mild cognitive impairment, with specific deficits in complex problem‐solving and memory.

**FIGURE 1:**
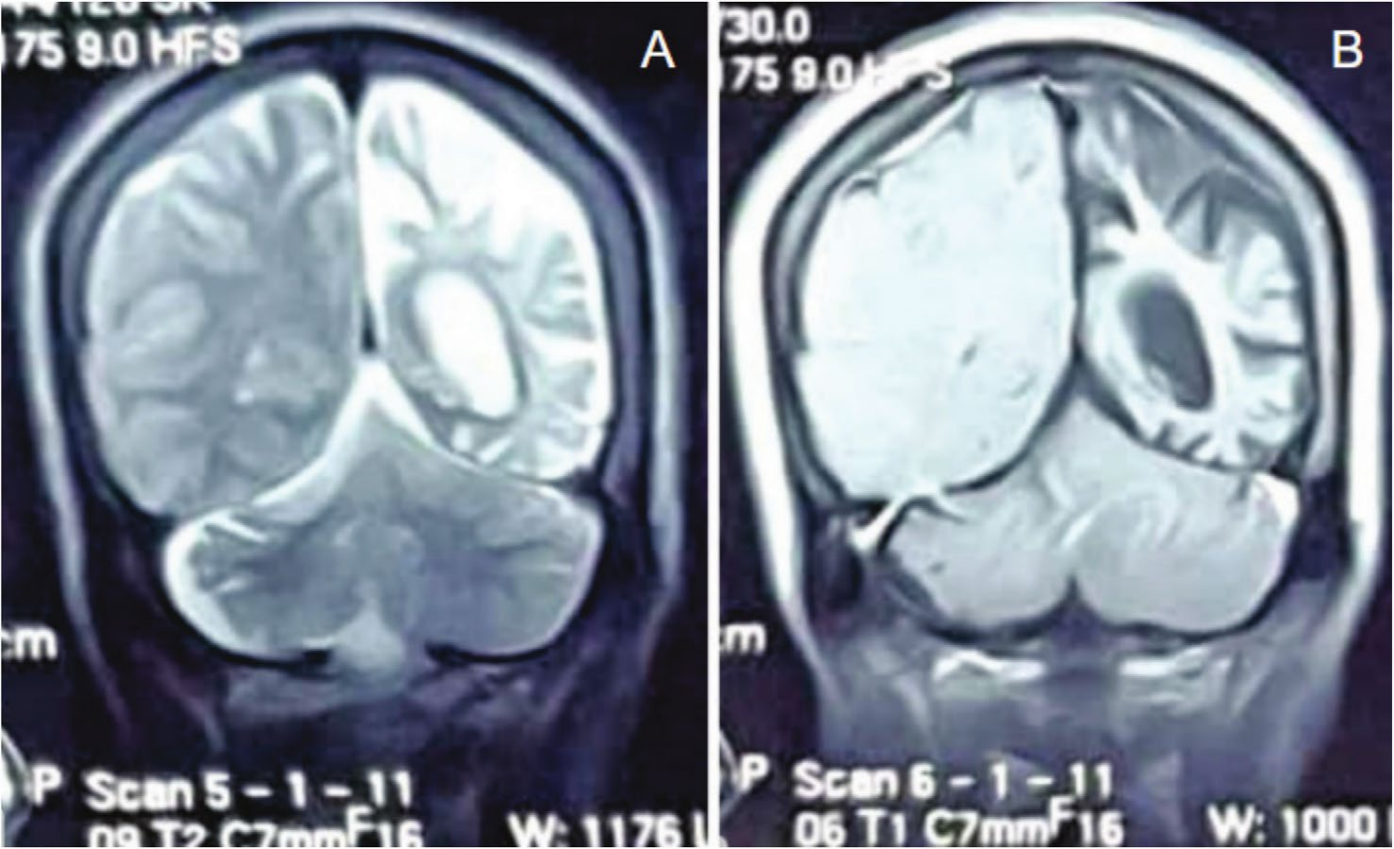
(A) Coronal T2‐weighted MRI showing left hemicerebral atrophy with ex‐vacuo dilatation of the left lateral ventricle. (B) Coronal T1‐weighted post‐contrast MRI displaying similar findings with no abnormal enhancement.

**FIGURE 2:**
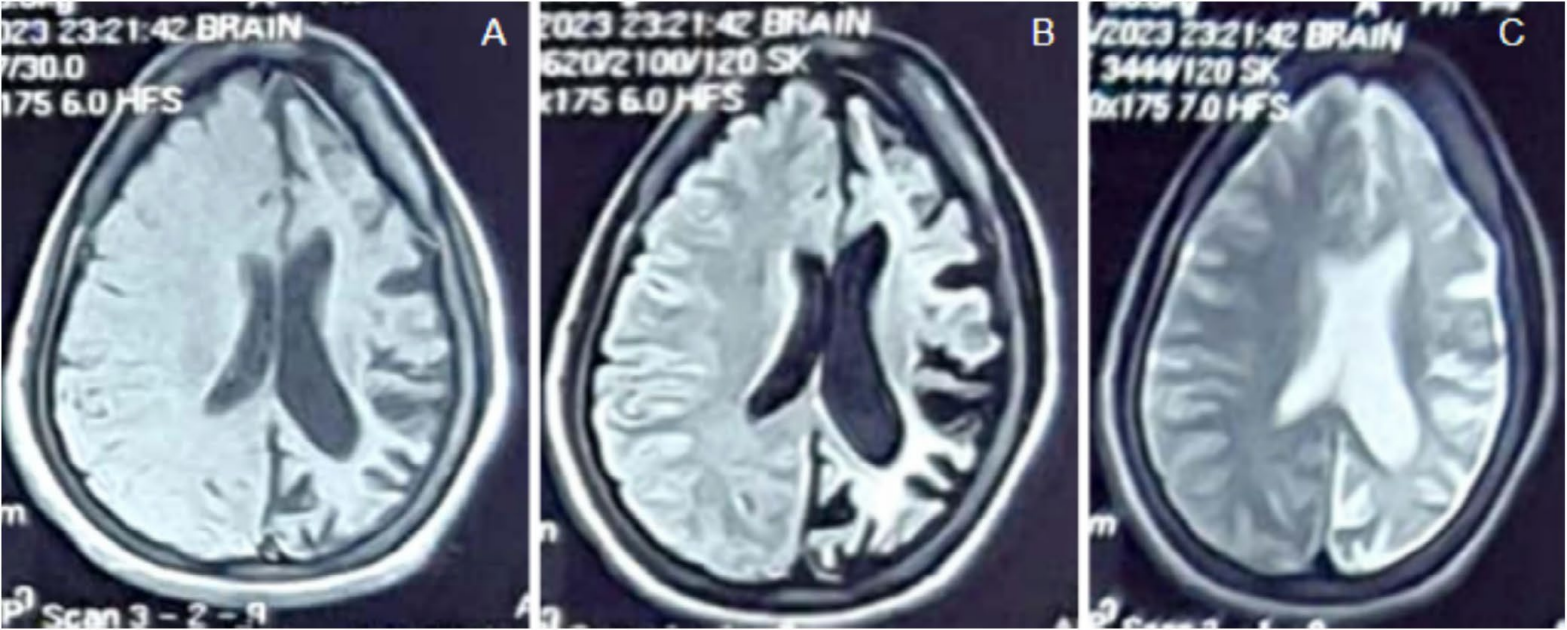
(A) Hemicerebral atrophy. (B) Ex‐vacuo dilatation of the ipsilateral lateral ventricle. (C) Ipsilateral calvarial thickening. Imaging findings demonstrate varying degrees of cerebral hemiatrophy with associated compensatory hypertrophy of the skull and sinuses.

An MRI of the brain showed ex‐vacuo dilatation of the ipsilateral lateral ventricle and left‐sided cerebral hemiatrophy (Figure 1). There was also dilatation of the frontal and sphenoid sinuses, thickening of the ipsilateral calvaria, and hyperpneumatization of the mastoid air cells (Figure 2). The cerebellum, internal capsule, and right basal ganglia appear intact. Lack of aberrant post‐contrast enhancement ruled out active inflammatory or neoplastic processes.

**FIGURE 3:**
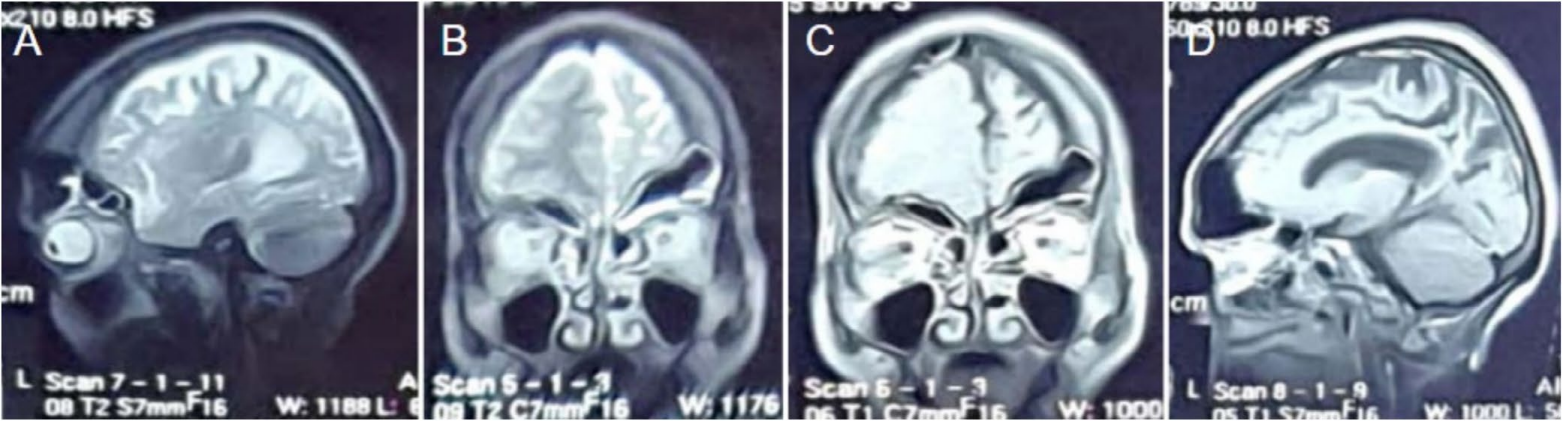
(A) T1 sagittal MRI showing hypertrophy of the left frontal and ethmoid sinuses with left calvarial thickening. (B) T2 coronal MRI demonstrating left hemicerebral atrophy. (C) T1 post‐contrast coronal MRI. (D) T1 post‐contrast sagittal MRI.

**FIGURE 4:**
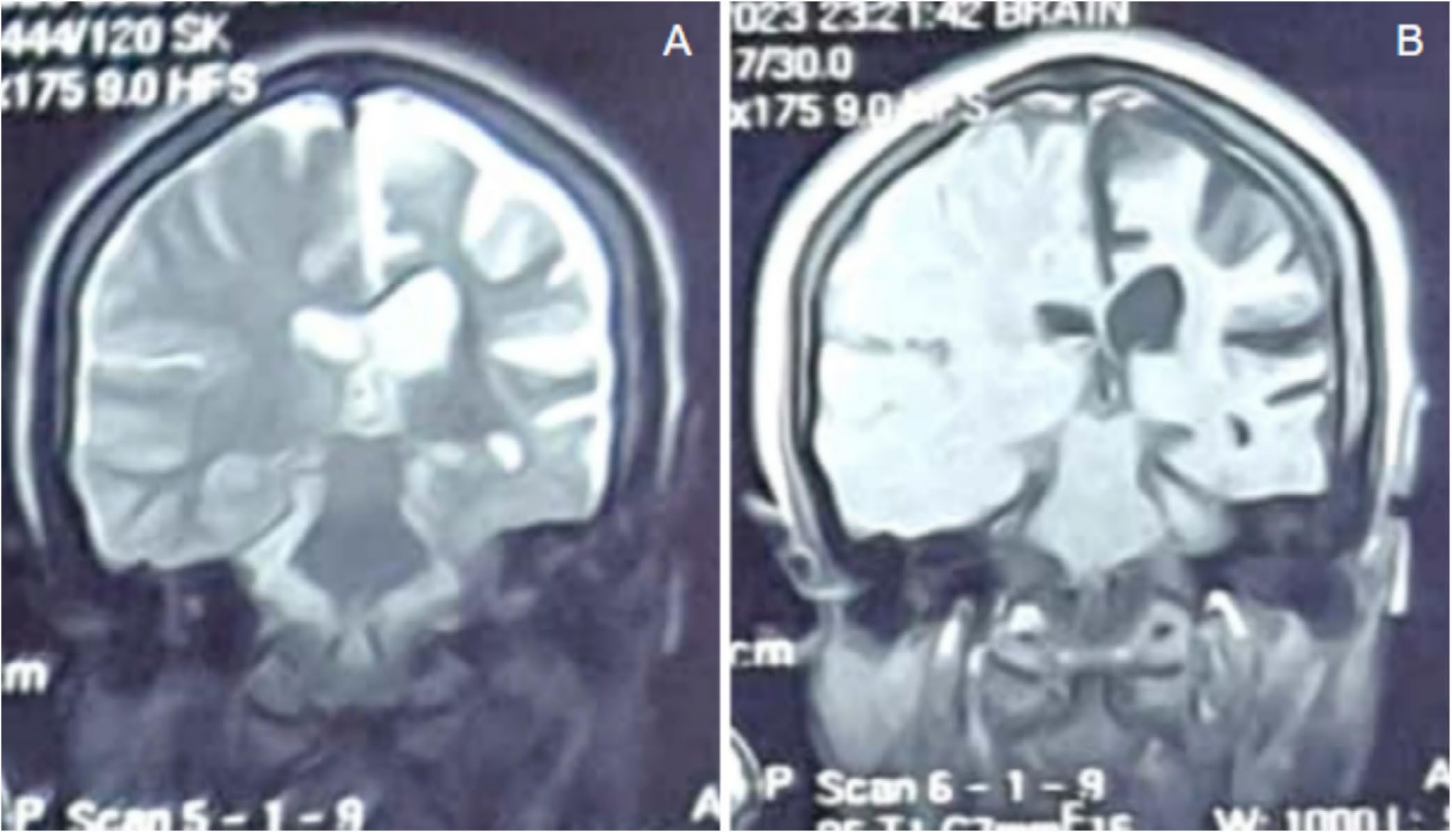
(A) T2 coronal MRI showing hyperpneumatization of left mastoid air cells with slight elevation of the left petrous ridge. (B) T1 post‐contrast coronal MRI demonstrating left lateral ventricle dilation and calvarial thickening.

MRI findings confirmed left‐sided cerebral hemiatrophy with calvarial thickening, hyperpneumatization of ipsilateral mastoid air cells, and compensatory sinus hypertrophy (Figures 3 and 4). Based on these findings, Dyke‐Davidoff‐Masson syndrome was diagnosed. The patient was referred for neurological follow‐up and physiotherapy to manage her motor impairments, and her antiepileptic medication was modified to better control her seizures. To evaluate the course of the condition and improve treatment approaches, long‐term surveillance was suggested.

## 3 Differential Diagnosis

Another factor to take into account is Sturge–Weber syndrome, which likewise involves unilateral brain involvement. Leptomeningeal angiomatosis and distinctive facial port‐wine stains, which are not present in DDMS, are indicative of this neurocutaneous condition. Furthermore, Sturge–Weber syndrome manifests as increasing calcifications in impacted brain areas that are visible on imaging tests.

Asymmetric cerebral atrophy, which resembles DDMS, can be caused by perinatal ischemia traumas. The typical compensatory bone and sinus enlargement associated with DDMS is typically absent in these patients, though. Another possible differential is hemimegalencephaly, a developmental abnormality marked by an enlarged rather than atrophic hemisphere. On neuroimaging, hemimegalencephaly can be distinguished from DDMS by its cortical thickness and excessive gyration.

In light of these factors, MRI is essential for distinguishing DDMS from other causes of unilateral cerebral atrophy. One characteristic that continues to help in diagnosis is the combination of sinus hypertrophy, compensatory skull thickening, and cerebral hemiatrophy.

## 4 Conclusion and Results

The patient was referred for neurological follow‐up and physiotherapy. Her antiepileptic medication regimen was adjusted to enhance seizure control. Long‐term surveillance was recommended to monitor disease progression and optimize symptom management. Early intervention in such cases improves mobility and quality of life.

## 5 Discussion

Dyke‐Davidoff‐Masson syndrome (DDMS) is a rare neurologic disorder first described in 1933 and characterized by cerebral hemiatrophy with secondary skull changes, including calvarial thickening and frontal and mastoid sinus hyperpneumatization [7]. The present case is in accord with classical imaging findings of DDMS, like left‐sided brain atrophy, ipsilateral ventricular dilatation, calvarial thickening, and frontal and mastoid sinus hyperpneumatization [8].

The etiopathogenesis of DDMS is traditionally categorized as acquired or congenital. Congenital types are usually caused by intrauterine vascular insults or brain developmental malformations [9]. Acquired DDMS, by contrast, is usually caused by postnatal brain insult such as trauma, infection, ischemic stroke, or febrile seizures in early life that persist for a prolonged duration [3].

Our patient presented with progressive seizures, right‐sided weakness, and impaired cognition—features characteristic of the most common clinical presentation of DDMS [10]. Seizures in DDMS are generally resistant to standard antiepileptic treatment and may require polytherapy or surgery in severe cases [11]. The motor deficits, particularly hemiparesis with spasticity and contractures in this case, are commonly progressive and have a significant role in producing disability [12].

DDMS diagnosis is radiologically directed. MRI would reveal unilateral cerebral atrophy, ipsilateral ventriculomegaly, and compensatory features such as skull thickening and hyperpneumatization of sinuses [8]. The findings above can distinguish DDMS from disorders that mimic its similar neurological presentation. Rasmussen's encephalitis, for example, can have unilateral atrophy and seizures but in the absence of DDMS's compensatory skull changes [11]. Sturge–Weber syndrome, a differential diagnosis, consists of leptomeningeal angiomatosis and characteristic facial port‐wine stains, both of which were absent in our patient [7].

For our patient, absence of post‐contrast enhancement on MRI further excluded inflammatory and neoplastic etiologies, making the diagnosis of DDMS more likely [3]. Ipsilateral hypertrophy of calvaria and sinus is pathognomonic and typically accompanies cerebral insult of early life [8].

Management of DDMS is multidisciplinary. Seizure is managed with antiepileptic drugs, although surgery such as hemispherectomy can be considered in resistant cases [11]. Physiotherapy also becomes significant in motor rehabilitation, particularly to manage spasticity and prevent fixed deformities due to contractures [12]. Cognitive therapy and follow‐up over a long period of time also play significant roles, particularly in intellectual impairment patients [3].

This case illustrates the importance of early neuroimaging in chronic, drug‐resistant seizures and unilateral motor deficit. Identification of the pathognomonic features of DDMS can guide diagnosis and encourage multidisciplinary intervention, perhaps to improve long‐term prognosis [9].

## Author Contributions

**Quang La:** conceptualization, data curation, formal analysis, investigation, methodology, project administration, resources, software, visualization, writing – original draft, writing – review and editing. **Aiman Baloch:** conceptualization, data curation, formal analysis, investigation, methodology, project administration, resources, software, supervision, writing – original draft, writing – review and editing. **Muhammad Ayub:** data curation, supervision, validation, writing – original draft, writing – review and editing. **Sobia Ahmed:** supervision, validation, writing – original draft, writing – review and editing. **Enoch Lue:** writing – original draft, writing – review and editing.

## Consent

Written informed consent was obtained from the patient to publish this report in accordance with the journal's patient consent policy.

## Conflicts of Interest

The authors declare no conflicts of interest.

## Data Availability

The data that support the findings of this study are available on request from the corresponding author. The data are not publicly available due to privacy or ethical restrictions.
